# Inhibition of the Glycine Receptor alpha 3 Function by Colchicine

**DOI:** 10.3389/fphar.2020.01143

**Published:** 2020-07-30

**Authors:** Carola Muñoz-Montesino, Carlos F. Burgos, Cesar O. Lara, Camila R. Riquelme, David Flaig, Victoria P. San Martin, Luis G. Aguayo, Jorge Fuentealba, Patricio A. Castro, Leonardo Guzmán, Gonzalo E. Yévenes, Gustavo Moraga-Cid

**Affiliations:** Departamento de Fisiología, Facultad de Ciencias Biológicas, Universidad de Concepción, Concepción, Chile

**Keywords:** glycine receptor, antagonist, colchicine, pentameric ligand-gated ion channel, pain

## Abstract

Colchicine is a plant alkaloid that is widely used as a therapeutic agent. It is widely accepted that colchicine reduces the production of inflammatory mediators mainly by altering cytoskeleton dynamics due to its microtubule polymerization inhibitory activity. However, other lines of evidence have shown that colchicine exerts direct actions on the function of ion channels, which are independent of cytoskeleton alterations. Colchicine is able to modify the function of several pentameric ligand-gated ion channels, including glycine receptors (GlyRs). Previous electrophysiological studies have shown that colchicine act as an antagonist of GlyRs composed by the *α*_1_ subunit. In addition, it was recently demonstrated that colchicine directly bind to the *α*_3_ subunit of GlyRs. Interestingly, other studies have shown a main role of *α*_3_GlyRs on chronic inflammatory pain. Nevertheless, the functional effects of colchicine on the *α*_3_GlyR function are still unknown. Here, by using electrophysiological techniques and bioinformatics, we show that colchicine inhibited the function of the *α*_3_GlyRs. Colchicine elicited concentration-dependent inhibitory effects on *α*_3_GlyRs at micromolar range and decreased the apparent affinity for glycine. Single-channel recordings show that the colchicine inhibition is associated with a decrease in the open probability of the ion channel. Molecular docking assays suggest that colchicine preferentially bind to the orthosteric site in the closed state of the ion channel. Altogether, our results suggest that colchicine is a competitive antagonist of the *α*_3_GlyRs.

## Introduction

Colchicine is a tricyclic alkaloid known for their effects over cytoskeleton, acting as a microtubule depolymerizing agent (Slobodnick et al., 2015). Colchicine has been used in a variety of illnesses, including rheumatic and cardiovascular diseases (Campbell et al., 2015; Slobodnick et al., 2015). By changing cytoskeleton dynamics through microtubule disruption, colchicine affects cellular processes including neutrophil extravasation, cytokine secretion among others events associated with inflammation (Dalbeth et al., 2014). Based on these properties, it has been approved by the FDA for the treatment of inflammatory diseases in 2009 (Center for Drug Evaluation and Research).

Several electrophysiological studies have shown that colchicine modulates neurotransmission and the function of pentameric ligand-gated ion channels (pLGICs) by altering the microtubule organization (Whatley et al., 1994; van Zundert et al., 2002; Whatley et al., 2002; Van Zundert et al., 2004). For example, alteration of microtubule stability by colchicine selectively impaired the function of the synaptic and extrasynaptic glycine receptors (GlyRs) in spinal neurons (van Zundert et al., 2002; van Zundert et al., 2004) and reduced the function and allosteric modulation of the *γ*-aminobutyric acid receptor type A (GABA_A_Rs) (Whatley et al., 1994; Whatley et al., 2002). Nonetheless, several works have reported the direct effects of colchicine on the function of inhibitory and excitatory pLGICs. Electrophysiological experiments performed in *Xenopus* oocytes have shown that colchicine acts as a competitive antagonist of GlyRs (Machu, 1998) and of GABA_A_Rs (Bueno and Leidenheimer, 1998). The inhibition of the glycine and GABA-activated currents was observed instantly and was concentration-dependent. Since the depolymerization of microtubules with colchicine takes at least 1.5 h to reach equilibrium at 30°C (Owellen et al., 1972), the inhibitory actions of colchicine on GlyRs and GABA_A_Rs were proposed to be independent of microtubule alterations. In addition, the effects of colchicine on GABA responses required no pre-incubation with colchicine (Whatley et al., 1994), and pre-incubation with the drug failed to enhanced its effect on GlyRs (Machu, 1998). Moreover, other microtubule-depolarizing agents failed to have similar effects over the ion channel function (Machu, 1998). On the other hand, further reports have shown that colchicine is able to modulate the 5-Hydroxytryptamine 3A receptor (5-HT_3A_R). Similar to GlyRs and GABA_A_Rs, the colchicine inhibition of 5-HT_3A_R function occurred in the absence of pre-incubation through microtubule-independent mechanisms (de Oliveira-Pierce et al., 2009).

Using biochemical techniques, a recent report has demonstrated that colchicine is able to bind homo-pentameric *α*_3_GlyRs (Zhou et al., 2018). This report is particularly interesting because colchicine is an effective treatment for the inflammatory pain in gout flares (Dalbeth et al., 2014). On the other hand, recent reports have shown that positive allosteric modulators of *α*_3_-containing GlyRs displayed analgesic effects in behavioral models of chronic pain (Acuña et al., 2016; Zeilhofer et al., 2018). These pieces of evidence suggest that colchicine may exert part of its therapeutic effects through the modulation of *α*_3_GlyRs. However, the effects of colchicine in the function of *α*_3_-containing GlyRs are still unknown. In this work, we combine *in silico* docking assays and electrophysiological recordings to evaluate the effects of colchicine in the function of homo-pentameric *α*_3_GlyRs.

## Material and Methods

### Cell Culture and Transfection

HEK 293 cells (CRL-1573; American Type Culture Collection, Manassas, VA, USA) were cultured using standard methods (Lara et al., 2019). The cells were transfected using XfectTM Transfection Reagent (Clontech, USA) using 1.0 μg of cDNA plasmid encoding the rat GlyR *α*_3_ subunit. Cell were co-transfected with a plasmid encoding EGFP (0.5 μg) to identify the transfected cells (Lara et al., 2019). All recordings were made 24–36 h after transfection.

### Electrophysiology

Glycine-evoked currents were recorded from EGFP-positive transfected HEK 293 cells in the whole-cell voltage-clamp configuration at room temperature (20–24°C) at a holding potential of −60 mV (Lara et al., 2019). Patch electrodes (3–4 mΩ) were pulled from borosilicate glass and were filled with (in mM): 120 CsCl, 8 EGTA, 10 HEPES (pH 7.4), 4 MgCl_2_, 0.5 GTP, and 2 ATP. The external solution contained (in mM) 140 NaCl, 5.4 KCl, 2.0 CaCl_2_, 1.0 MgCl_2_, 10 HEPES (pH 7.4), and 10 glucose. Whole-cell recordings were performed with an Axoclamp 200B amplifier (Molecular Devices, USA) and acquired using Clampex 10.1 or Axopatch 10.0 software. Data analysis was performed off-line using Clampfit 10.1 (Axon Instruments, Sunnyvale, CA, USA). Exogenous glycine-evoked currents were obtained using a manually applied pulse (3–4 s) of the agonist and an outlet tube (200 μm ID) of a custom-designed gravity-fed microperfusion system positioned 50–100 μm from the recorded cell. The methodologies employed for the single channel recordings of *α*_3_GlyRs in the cell-attached configuration have been previously published (Marabelli et al., 2013; Lara et al., 2019). The patch pipettes had tip resistances of 10–20 mΩ and were manually fire polished in a microforge (Narishige, Japan). The data were filtered (1-kHz low-pass 8-pole Butterworth) and acquired at 5–20 kHz using an Axopatch 200B amplifier and a 1322A Digidata (Axon Instruments, Union City, CA). Data was acquired using pClamp software and analyzed off-line with Clampfit 10.1 (Axon Instruments, Union City, CA). Colchicine stock was prepared in high purity distilled water and subsequently diluted into the recording solution on the day of the experiment. In whole-cell experiments colchicine was co-applied with glycine using a manually applied pulse (1–2 s). In cell-attached recordings, colchicine was applied to the intra-pipette solution together with glycine. Colchicine was obtained from AK Science (CA, USA). All other reagents were from Sigma-Aldrich (St. Louis, MO, USA).

### Molecular Docking Simulations

Protein-ligand docking was performed using the structures of *α*_3_GlyRs in open/closed conformations from the Protein DataBank (PDB ID:5CFB, 5TIO) (Huang et al., 2007; Huang et al., 2017). The structures of colchicine, strychnine, and glycine were obtained from the PubChem database (CID: 26719, 441071, 750) and prepared using LigPrep (Schrödinger, LLC, New York, NY, 2016) before docking simulations (Irwin et al., 2012). Initially, free docking protein-ligands were performed using Autodock Vina (Trott and Olson, 2010) in which the extracelullar domain (ECD) of the *α*_3_GlyRs was used as the protein target. The generated complexes were ranked based on the affinity constants calculated by the same program. Subsequently, site-directed docking was created with Glide (Schrödinger, LLC, New York, NY, 2016) using a receptor grid centered on the amino acids that form the binding site defined in the previous step and an extra-precision (XP) configuration. Analysis of the interface GlyR-molecules included structural and energetic parameters performed by the same software. Additionally, a theoretical ΔG bind was calculated by an energy calculation MM-GBSA using Prime (Schrödinger, LLC, New York, NY, 2016). All images were created using PyMol (version 1.5, DeLano Scientific LLC).

### Data Analysis

All values were expressed as mean ± s.e.m. of normalized agonist-activated currents. P < 0.05 was considered statistically significant. For statistical analyses, at least six cells were analyzed per condition. All the statistical analyses and plots were performed with MicroCal Origin 8.0 (Northampton, MA, USA).

## Results

**Figure 1:**
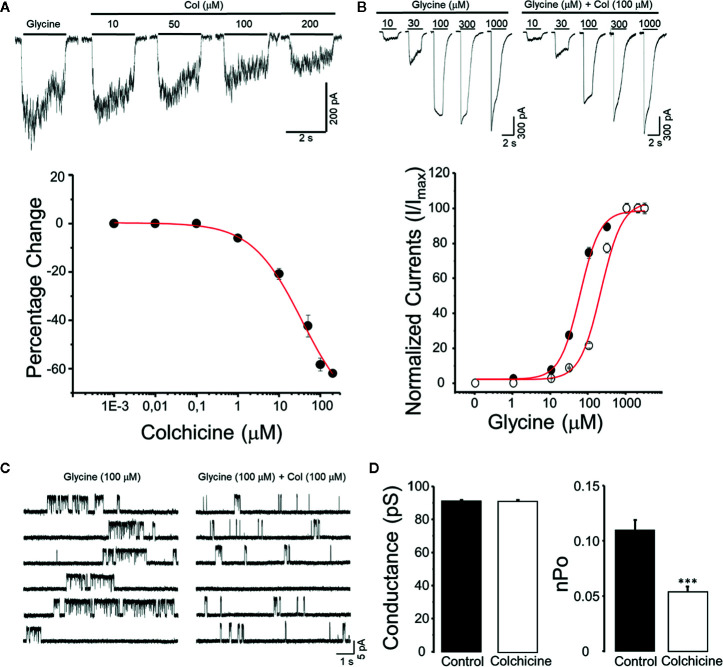
Functional modulation of *α*_3_GlyRs by colchicine. **(A)** The panel shows typical whole-cell current traces recorded in HEK293 cells expressing *α*_3_GlyRs activated by glycine 30 μM before and during the application of colchicine (Col) (10, 50, 100, and 200 μM). The graph summarizes the percentage of inhibition of the glycine-evoked currents in a concentration-response fashion. **(B)** The panel shows current traces recorded in HEK293 cells expressing *α*_3_GlyRs activated by glycine (1–1,000 μM) in the absence (left) or presence (right) of colchicine (Col 100 μM). The plot summarizes the glycine concentration-response curve obtained in the absence (black circles) or presence (white circles) of colchicine (100 μM). **(C)** Single-channel activity recorded in cell-attached configuration from cells expressing *α*_3_GlyR before and in the presence of 100 μM of colchicine. **(D)** The graphs show that colchicine did not modified the main unitary conductance but significantly decreased the open probability (nPo) of the *α*_3_GlyRs (***P < 0.001, paired Student t-Test).

To test the hypothesis that colchicine modulates directly the function of GlyRs, concentration response curves were generated from HEK293 expressing *α*_3_GlyRs. All these experiments were performed in the absence of any pre-incubation with colchicine. The co-application of colchicine produced a rapid inhibition of glycine-activated currents (tested at an EC_10–15_ concentration; 30–50 μM). The alkaloid exerted inhibitory effects in concentrations ranging from 1 to 200 μM (**Figure 1A**). The concentration producing the 50% inhibition (IC_50_) was 24 ± 7 μM, with a maximal inhibition of 39 ± 2% at 200 μM (n = 8). Interestingly, the colchicine potency observed in the *α*_3_GlyRs was not different from the IC_50_ obtained in the *α*_1_GlyRs (25 ± 6 μM, **Supplementary Figure S1**). To obtain additional insights on the nature of this inhibition, glycine concentration–response curves were generated in the absence or presence of 100 μM of colchicine (**Figure 1B**). A right-shift in the glycine concentration curve was observed in presence of colchicine, changing the apparent affinity from 56 ± 4 μM to 253 ± 29 μM (n = 8; p < 0.01). An estimated K_i_ value of 15 ± 3 μM in the presence of 200 μM of colchicine was calculated using the Cheng–Prusoff equation (Cer et al., 2009). To investigate further the mechanisms underlying the ion channel modulation by colchicine, we performed single-channel recordings in the cell-attached configuration (**Figures 1C, D**). No spontaneous activity was observed when patches were perfused with glycine-free solution (>1 min). However, glycine 100 μM triggered clusters of active periods (**Figure 1C**). The mean current amplitude at +60 mV calculated from the amplitude histogram was 5.5 ± 0.08 pA. The main single conductance (92 ± 2 pS) was determined by current–voltage relationship over a range of voltages from −80 mV to +80 mV as an average from three to five patches (**Figure 1D**). The application of colchicine (100 μM) to membrane patches obtained in the same control cells did not change the main current amplitude (5.4 ± 0.06 pA) and main conductance (91 ± 2 pS (n = 6; p = 0.66). In agreement with our data obtained from whole-cell experiments, colchicine significantly reduced the normalized open probability (nPo) by 47.3% (0.11 ± 0.09 in control conditions *v/s* 0.056 ± 0.04 in colchicine conditions (p < 0.01, paired t-test). Altogether, these results support a direct inhibition of the channel function by colchicine. Moreover, these data are consistent with a competitive antagonism of colchicine on the *α*_3_GlyRs.

**Figure 2:**
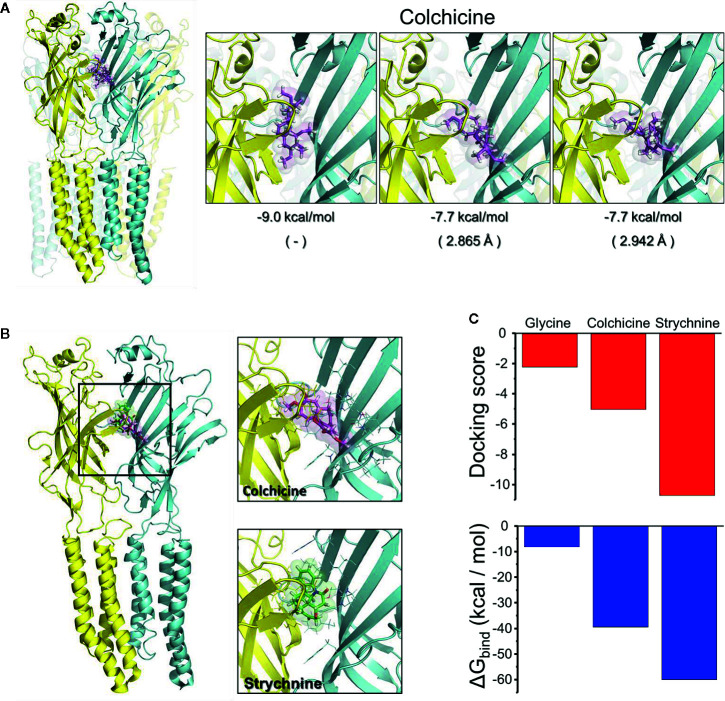
Binding prediction of colchicine on the *α*_3_GlyR structure. **(A)** Representative interaction modes of the free docking between the ECD of *α*_3_GlyR and colchicine. All chains are identical and were colored in cyan and yellow to facilitate the identification of intersubunit regions. **(B)** Binding of colchicine to the orthosteric site of *α*_3_GlyR predicted by Glide. For comparison, the binding of strychnine is also shown. **(C)** The graphs present the docking scores and the theoretical ΔG bind of interaction of colchicine and strychnine with *α*_3_GlyRs in the closed state. The value obtained from the docking with glycine under similar conditions has been added as reference.

We next performed *in silico* analysis of the interaction of *α*_3_GlyRs with colchicine using the crystal structure of *α*_3_GlyRs (PDB: 5CFB) as a template (**Figure 2**). We first performed a free docking protein-ligand using the ECD of *α*_3_GlyRs as a target region. Under this condition, colchicine showed a preference for the closed state of the *α*_3_GlyR, generating a series of complexes centered at the orthosteric binding site with slight differences between the orientations of the molecule (**Figure 2A**). We next restricted the docking analyses to the orthosteric site. The analyses revealed that colchicine binds to the closed state of *α*_3_GlyRs (**Figure 2B**), reaching a docking score of −5.05 and a predicted ΔG bind of −39.48 kcal/mol. The values were in the range to those showed by glycine and strychnine, suggesting a functional interaction (**Figure 2C**). The *α*_3_GlyRs–colchicine interface showed the formation of two H-bonds from amino acids Q177 (+) and T204 (−), together with a π-cation interaction with R65 (+) that contributes to the stabilization of the complex (**Supplementary Figure S2**). No *α*_3_GlyRs–colchicine complexes were observed when the open conformation of *α*_3_GlyRs was tested. Thus, our *in silico* studies match well with the functional data obtained in our electrophysiological recordings, suggesting that colchicine modulate the *α*_3_GlyRs function by direct binding to the orthosteric site, in a competitive manner.

## Discussion

Colchicine has been used for many years as a therapeutic agent. Its uses also include undesired effects, such as gastrointestinal disturbances and neutropenia. Traditionally, the mechanism by which colchicine affects the cell function has been linked to the inhibition of microtubule polymerization. Since the cytoskeleton controls many aspects of the cell physiology, such as migration and intracellular signaling, the capacity of colchicine as an anti-inflammatory agent has been associated with this specific action (Angelidis et al., 2018). Nevertheless, other studies have shown that colchicine exerts direct actions on ion channels, including GlyRs composed of *α*1 and *α*2 (Bueno and Leidenheimer, 1998; Machu, 1998; de Oliveira-Pierce et al., 2009). Interestingly, using a biochemical approach, a recent report demonstrated that colchicine in complex with biotin binds directly to *α*_3_GlyRs (Zhou et al., 2018). The authors thus proposed that the colchicine–*α*_3_GlyRs interaction may explain the mitigating effects of colchicine on inflammatory pain, especially the one caused by the deposition of monosodium urate crystal in gouty arthritis disease. Interestingly, these pieces of evidence are in line with experimental data showing the relevance of the *α*_3_GlyRs on chronic pain of inflammatory origin and the ability of positive allosteric modulators to exert analgesic effects in behavioral models of chronic pain (Ahmadi et al., 2002; Harvey et al., 2004; Acuña et al., 2016; Zeilhofer et al., 2018). However, the effects of colchicine on the *α*_3_GlyRs function were not described. The present work characterized the *α*_3_GlyRs–colchicine functional interaction by electrophysiology and bioinformatics. Our electrophysiological studies show that the function of the *α*_3_GlyRs is inhibited by colchicine at micromolar range (1–200 μM). The glycine-activated current inhibition was not significantly different to those obtained using *α*_1_GlyRs, in which colchicine was described as a competitive antagonist in a previous study (Machu, 1998). Single-channel analysis showed that colchicine reduced the ion channel open probability without changes in the conductance. Moreover, molecular modeling and *in silico* docking simulations based on the crystal structure of *α*_3_GlyRs (Huang et al., 2017) showed a favorable binding of colchicine to the orthosteric site. Interestingly, colchicine has a higher preference for the orthosteric site in the closed conformation. However, since our data suggest that colchicine is a competitive antagonist of the *α*_3_GlyRs, our results suggest that the analgesic effects of colchicine in inflammatory pain are possibly not linked to an enhanced *α*_3_GlyRs activity. In addition, the comparison between the plasmatic concentrations of colchicine from patients (0.6–9.5 ng/ml) (Berkun et al., 2012) and the concentrations required to inhibit the GlyR function reported here makes it difficult to suggest that the modulation of GlyRs is a relevant colchicine target in humans.

## Conclusion

The present work defines the modulatory effects of colchicine on homomeric *α*_3_GlyRs. Our results provide novel functional information regarding the direct interaction of colchicine with GlyRs, suggesting a molecular mechanism associated with a competitive inhibition at the orthosteric site. Although our experiments does not rule out a possible relevance of *α*_3_GlyRs on the analgesic actions of colchicine in inflammatory pain (especially in the context of gouty arthritis), our results at least confirm that GlyRs are targets of the alkaloid colchicine at the functional level. Further behavioral and functional experiments are necessary to clarify whether the effects of colchicine on GlyRs are relevant players on the beneficial effects of the alkaloid in inflammatory pain conditions.

## Data Availability Statement

All datasets generated for this study are included in the article/**Supplementary Material**.

## Author Contributions

CM-M, COL, CRR,DF and VPSM performed the experiments and data analysis. LGA, JF, PAC, LG, GEY and GM-C designed the research and contribute with analytical tools. CFB and CRR performed in silico analysis. CM-M, GEY and GM-C wrote the paper. All authors read and approved the submitted version.

## Funding

This work was supported by grant FONDECYT 1160851 and VRID 219.033.111-INV. (to GM-C), FONDECYT 1170252 (to GEY) and FONDECYT 3170108 (to CFB). COL and VPSM were supported by CONICYT doctoral fellowship 21171549 and 21201722 respectively. CRR was supported by CONICYT master fellowship 22200510.

## Conflict of Interest

The authors declare that the research was conducted in the absence of any commercial or financial relationships that could be construed as a potential conflict of interest.

## Supplementary Material

Figure S1 Functional modulation of *α*_1_GlyRs by colchicine. **(A)** The panel shows typical whole-cell current traces recorded in HEK293 cells expressing *α*1GlyRs activated by glycine 20 μM before and during the application of colchicine (Col) (10, 50, 100, and 200 μM). **(B)** The graph summarizes the percentage of inhibition of the glycine-evoked currents in a dose–response fashion (IC_50_ = 25 ± 6 μM, n = 6).

Figure S2 Ligand interaction diagrams of colchicine with the ECD of *α*_3_GlyRs. Schematic representation of the amino acids involved in the binding of colchicine with the ECD of *α*_3_GlyR in closed-state conformation. All interactions detected in the *α*_3_GlyR-colchicine interface are located in the inner box with a cutoff of 4 Å from the receptor. In the two H-bonds formed, T204 and Q177 act as hydrogen-bond donors. For its part, R65 interacts with an aromatic ring of colchicine, an electron-rich π group, to create a pi–cation interaction. The analysis was performed using Maestro (Schrödinger, LLC, NY, 2018).
