# Supplementary figures and images for: Inhibition of the Glycine Receptor alpha 3 Function by Colchicine
Source: Front Pharmacol. 2020 Jul 30;11:1143. doi: 10.3389/fphar.2020.01143 (PMC7438739; doi:10.3389/fphar.2020.01143)

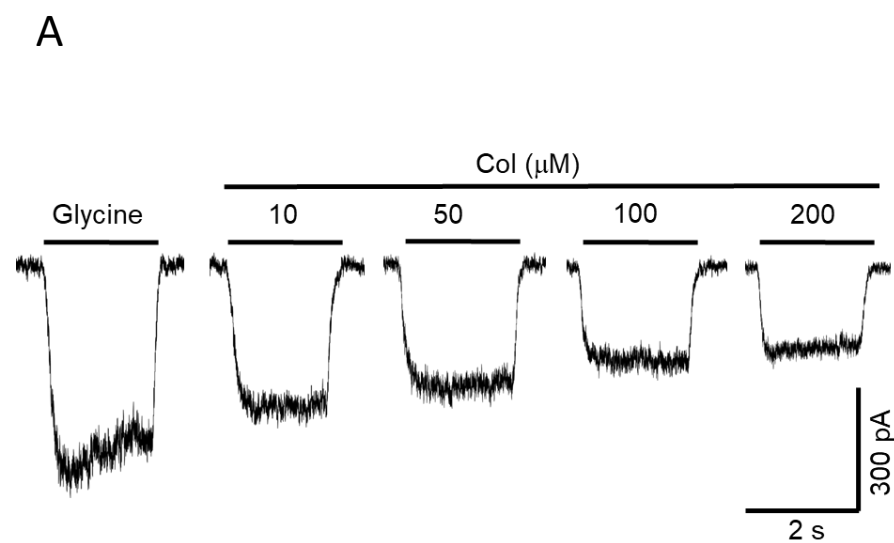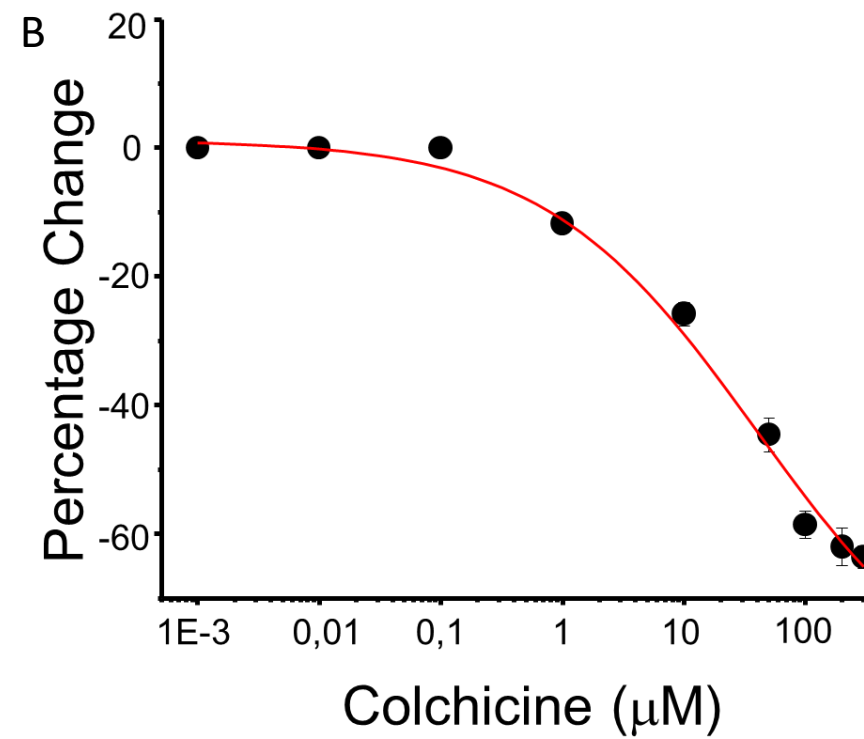

Figure S1

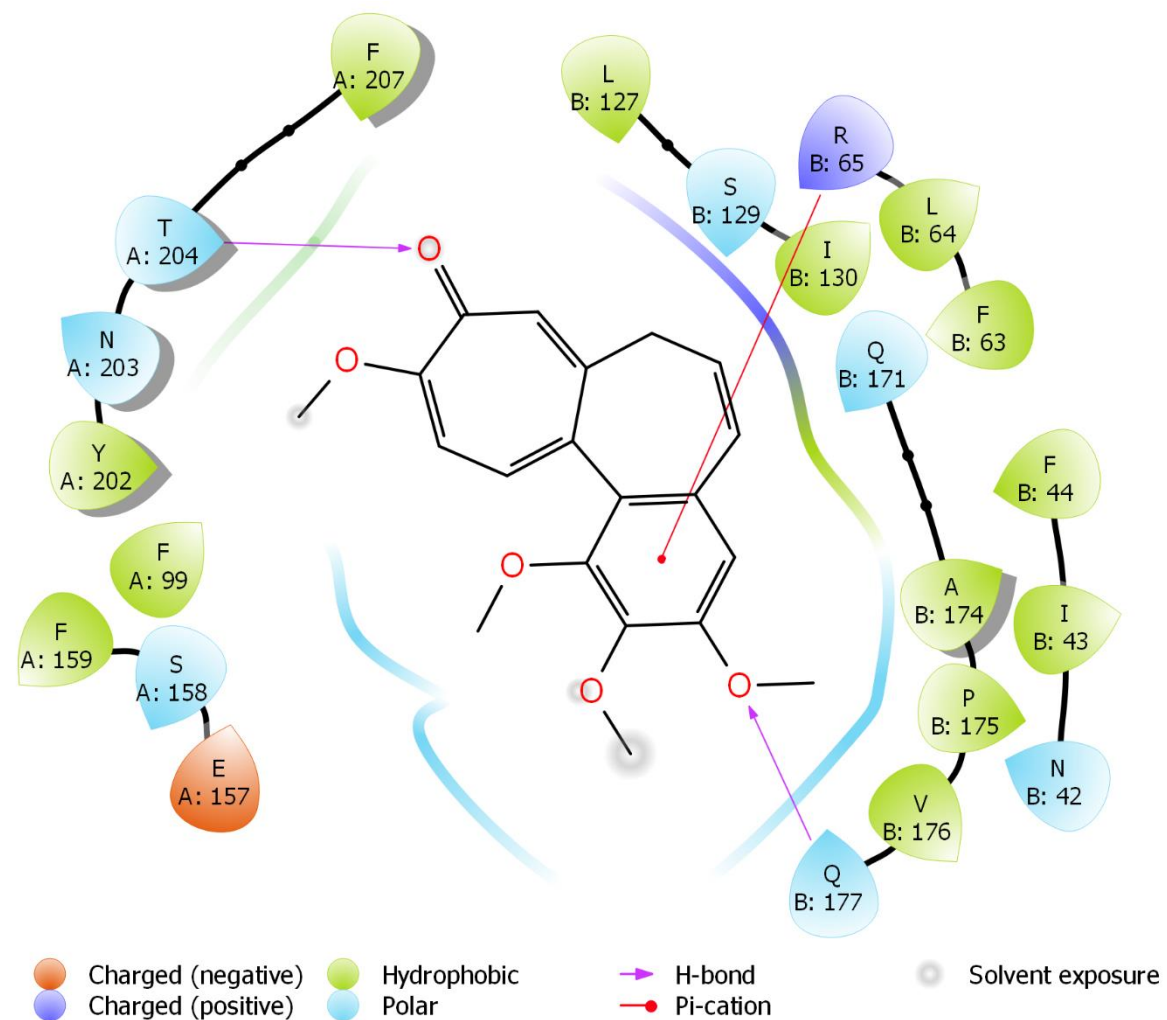

Figure S2

Supplement: Figure S1 — Functional modulation of α 1GlyRs by colchicine. (A) The panel shows typical whole-cell current traces recorded in HEK293 cells expressing α1GlyRs activated by glycine 20 μM before and during the application of colchicine (Col) (10, 50, 100, and 200 μM). (B) The graph summarizes the percentage of inhibition of the glycine-evoked currents in a dose–response fashion (IC50 = 25 ± 6 μM, n = 6). [file DataSheet_1.pdf]
